# Predictions of Preterm Birth from Early Pregnancy Characteristics: Born in Guangzhou Cohort Study

**DOI:** 10.3390/jcm7080185

**Published:** 2018-07-27

**Authors:** Jian-Rong He, Rema Ramakrishnan, Yu-Mian Lai, Wei-Dong Li, Xuan Zhao, Yan Hu, Nian-Nian Chen, Fang Hu, Jin-Hua Lu, Xue-Ling Wei, Ming-Yang Yuan, Song-Ying Shen, Lan Qiu, Qiao-Zhu Chen, Cui-Yue Hu, Kar Keung Cheng, Ben Willem J. Mol, Hui-Min Xia, Xiu Qiu

**Affiliations:** 1 Division of Birth Cohort Study, Guangzhou Women and Children’s Medical Center, Guangzhou Medical University, Guangzhou 510623, China; jianrong.he@bigcs.org (J.-R.H.); weidong.li@bigcs.org (W.-D.L.); xuan.zhao@bigcs.org (X.Z.); yan.hu@bigcs.org (Y.H.); niannian.chen@bigcs.org (N.-N.C.); fang.hu@bigcs.org (F.H.); jinhua.lu@bigcs.org (J.-H.L.); xueling.wei@bigcs.org (X.-L.W.); mingyang.yuan@bigcs.org (M.-Y.Y.); songying.shen@bigcs.org (S.-Y.S.); lan.qiu@bigcs.org (L.Q.); cuiyue.hu@gwcmc.org (C.-Y.H.); huimin.xia@bigcs.org (H.-M.X.); 2 Department of Obstetrics and Gynecology, Guangzhou Women and Children Medical Center, Guangzhou Medical University, Guangzhou 510623, China; yumian.lai@bigcs.org (Y.-M.L.); qiaozhu.chen@bigcs.org (Q.-Z.C.); 3 Nuffield Department of Women’s & Reproductive Health, University of Oxford, Oxford OX3 9DU, UK; rema.ramakrishnan@wrh.ox.ac.uk; 4 Department of Woman and Child Health Care, Guangzhou Women and Children’s Medical Center, Guangzhou Medical University, Guangzhou 510623, China; 5 Institute of Applied Health Research, University of Birmingham, Birmingham B15 2TT, UK; k.k.cheng@bham.ac.uk; 6 Department of Obstetrics and Gynecology, Monash University, Clayton, Victoria 3204, Australia; ben.mol@monash.edu; 7 Department of Neonatal Surgery, Guangzhou Women and Children’s Medical Center, Guangzhou Medical University, Guangzhou 510623, China

**Keywords:** preterm birth, prediction, early pregnancy, Chinese, external validation

## Abstract

Preterm birth (PTB, <37 weeks) is the leading cause of death in children <5 years of age. Early risk prediction for PTB would enable early monitoring and intervention. However, such prediction models have been rarely reported, especially in low- and middle-income areas. We used data on a number of easily accessible predictors during early pregnancy from 9044 women in Born in Guangzhou Cohort Study, China to generate prediction models for overall PTB and spontaneous, iatrogenic, late (34–36 weeks), and early (<34 weeks) PTB. Models were constructed using the Cox proportional hazard model, and their performance was evaluated by Harrell’s c and D statistics and calibration plot. We further performed a systematic review to identify published models and validated them in our population. Our new prediction models had moderate discrimination, with Harrell’s c statistics ranging from 0.60–0.66 for overall and subtypes of PTB. Significant predictors included maternal age, height, history of preterm delivery, amount of vaginal bleeding, folic acid intake before pregnancy, and passive smoking during pregnancy. Calibration plots showed good fit for all models except for early PTB. We validated three published models, all of which were from studies conducted in high-income countries; the area under receiver operating characteristic for these models ranged from 0.50 to 0.56. Based on early pregnancy characteristics, our models have moderate predictive ability for PTB. Future studies should consider inclusion of laboratory markers for the prediction of PTB.

## 1. Introduction

Preterm birth (PTB), the leading cause of death in children under five years of age [1,2], is associated with an increased risk for various short- and long-term adverse outcomes [3]. Numerous studies have attempted to predict the risk of PTB in pregnant women [4], but there is no prediction model available that is accurate enough to apply to clinical practice. Most studies have focused on predictors during the second or third trimester (e.g., cervical length or fetal fibronectin) [5]. However, these predictors have been proved to be accurate only in a high-risk population and can only predict intermediate risk of PTB [5]. Unfortunately, most women who deliver prematurely have no obvious risk factors and over half of PTBs occur in low-risk pregnancies, suggesting the limited utility of the use of cervical length or fetal fibronectin in a general population [6]. Also, prediction of PTB at a later stage in pregnancy limits the potential for early intervention, while prediction in early pregnancy would enable early monitoring and intervention.

Using easily accessible data to predict PTB appears to be attractive in low- and middle-income areas because of limited resources. However, very few models have been published. Of importance also is the substantial variation in PTB incidence worldwide, suggesting disparities in exposure to psychosocial, sociodemographic, and medical risk factors, and genetic differences [7,8,9]. Therefore, there is a need to construct and compare PTB prediction models in different populations. Despite a moderate incidence of PTB (7.1%) [10], the absolute number of preterm births in China is the second highest in the world, emphasizing an urgent need for prevention. As far as we know, there are only two published studies about PTB prediction in a Chinese population, and neither of them have focused on prediction in early pregnancy [11,12].

We aimed to assess the predictive capacity of pre-pregnancy characteristics, pregnancy conditions, and modifiable factors for PTB in a general population of pregnant women. Further, we conducted a systematic review to identify published models that have used easily accessible predictors for PTB and validated these models in our study population.

## 2. Materials and Methods

### 2.1. Subjects

This study was part of the Born in Guangzhou Cohort Study (BIGCS), a prospective study conducted by the Guangzhou Women and Children’s Medical Center (GWCMC), China. BIGCS aims to investigate the short- and long-term effects of characteristics during pregnancy and early life on the health of a young generation in China. The protocols of BIGCS were approved by the Institutional Ethics Committee of GWCMC (2013053107). All participants gave written informed consent. The recruitment strategy has been described elsewhere [13,14].

Pregnant women who attended their first routine antenatal examination (usually around week 16) at two campuses of GWCMC, resided within Guangzhou, and intended to stay in Guangzhou for at least three years were eligible for BIGCS. Maternal exposures were assessed via self-completed questionnaires. The first (Q1), second (Q2), and third (Q3) questionnaires were administered at recruitment (around 16 weeks) and at gestational ages of 24–27 weeks and 35–38 weeks, respectively. The questionnaires acquired demographic and socioeconomic information and data on exposures in the workplace and at home, personal lifestyle, medical histories, and health status before and during pregnancy. Gestational age was confirmed by ultrasound examination in the first- or early second-trimester.

### 2.2. Predictors

We used easily accessible data on maternal characteristics obtained at recruitment (around 16 weeks) as potential predictors. These included pre-pregnancy factors (socio-demographic characteristics, obstetric and disease history), pregnancy conditions, and modifiable factors. Socio-demographic variables included age at recruitment (continuous), educational level (middle school or below, technical/vocational college, undergraduate, postgraduate), monthly income (≤1500, 1501–4500, 4501–9000, or ≥9001 (Chinese Yuan), height (continuous), and pre-pregnancy body mass index (BMI, continuous). Variables of obstetric and disease history included gravidity (1 vs. >1), parity (nulliparous or multiparous), previous preterm delivery (no or yes), planned pregnancy (yes or no), diabetes/hypertension before pregnancy (no or yes), thyroid disease before pregnancy (no or yes), family history of diabetes (no or yes), family history of hypertension (no or yes), and family history of heart disease (no or yes). Variables of pregnancy conditions included timing of any vaginal bleeding during pregnancy (never, <13 weeks, or ≥13 weeks), amount of vaginal bleeding (none, mild, moderate or severe), and anxiety (continuous score) and depression (continuous score) during pregnancy. Variables of modifiable factors included working posture (not working, sitting, standing, walking or others), smoking during periconception period (no or yes), passive smoking before pregnancy (no or yes), passive smoking during pregnancy (no or yes), folic acid intake before pregnancy (no or yes), and folic acid intake during pregnancy (no or yes). We assessed depression and anxiety at recruitment by a 20-item self-rating depression scale (SDS) [15] and 20-item self-rating anxiety scale (SAS) [16], respectively. Both instruments have been validated in the Chinese population [15,16].

### 2.3. Outcomes

The primary outcome was PTB, defined as birth at less than 37 weeks of gestation. We also studied spontaneous PTB and iatrogenic PTB separately. Spontaneous PTB was defined as births following spontaneous preterm labor and/or preterm premature rupture of the membranes (PPROM) regardless of delivery mode (vaginal or caesarean section [17]). Iatrogenic PTB was defined as births following induced labor or pre-labor caesarean delivery [17]. We also conducted analyses for late (delivered at 34–36 weeks) and early (delivered at less than 34 weeks) PTB.

### 2.4. Model Development

We used a Cox proportional hazards regression model to construct the prediction model with preterm birth as the event and gestational age at birth as the time scale. We used Martingale residuals to examine linearity between continuous variables (maternal age, height, pre-pregnancy BMI, anxiety, and depression) and the risk of overall PTB. All continuous variables had a linear relationship with PTB. We used log (-log) survival plots, Schoenfeld residuals, and interaction term with time to assess the proportionality constant assumption. Parity was the only variable that violated this assumption, for early PTB. Therefore for early PTB, we constructed models stratified by parity (nulliparous and multiparous).

We constructed a three-step model for the prediction of PTB. The first model (Model 1) was based on pre-pregnancy factors. The second model (Model 2) utilized pre-pregnancy factors and pregnancy conditions. The final model (Model 3) included pre-pregnancy factors, pregnancy conditions and modifiable factors. The variables retained in the final models were selected using backward elimination. Based on the biological plausibility, we examined the interactions between each potential predictor and maternal age; education and anxiety, depression, folic acid intake before/during pregnancy, smoking during periconception period, and passive smoking before/during pregnancy; and folic acid intake before/during pregnancy and history of previous preterm delivery and smoking during periconception period. None of interaction terms were statistically significant (*p* values > 0.10).

Separate models were generated for overall, spontaneous, iatrogenic, late and early PTB. For overall, spontaneous, iatrogenic, and late PTB models, we censored term deliveries (≥37 weeks) at 37 weeks; for models of early PTB, we censored deliveries at ≥34 weeks. When modeling for late PTB, we excluded early PTBs. The hazard ratios (HR) and their 95% confidence intervals (CIs) for variables retained in the model were calculated. Since we observed that the timing and the amount of vaginal bleeding were correlated, we only included the amount of vaginal bleeding (and not the timing of it) in the development of the model because of its greater contribution.

We used multiple imputation by fully conditional specification to account for missing data. For each model, predictors that remained in at least three out of five imputed datasets were selected as the final predictors. We used Rubin’s rules to combine the regression coefficients and standard errors of final predictors and obtain the final model. Model performance was evaluated using discrimination and calibration. We used Harrell’s c statistic, a measure similar to the area under a receiver operating characteristic curve (AUC) and Somer’s D statistic, to evaluate the discriminative ability of each model. Harrell’s c and Somer’s D statistics with 95% confidence interval (CI) were estimated for each imputed dataset and then combined using Rubin’s rules. Goodness of fit was assessed using a calibration plot that shows the agreement between the predicted and observed risks of PTB [18,19]. We used the regression coefficients in the final model and the baseline survivor function in Cox regression model (by 37 weeks for overall PTB, spontaneous PTB, iatrogenic PTB, and late PTB and by 34 weeks for early PTB) to calculate the predicted risk of PTB for each pregnant women in each dataset. We then calculated the mean predicted risk of each pregnant women by averaging the predicted risks across the imputed datasets. The observed risk was obtained by using the Kaplan-Meier estimate. Finally, the Nam and D’Agostino test was used to compare the mean predicted and observed risks by deciles of predicted probability using the SAS macro provided by Demler [20]. The analysis was repeated by parity (nulliparous and multiparous). All analyses were performed using SAS statistical software version 9.3 (SAS Institute Inc., Cary, NC, USA) except the computation of Harrell’s c and Somer’s D statistics with 95% CI; the somersD package in STATA software (version14.0, StataCorp LP, College Station, TX, USA) was utilized for this purpose [18].

### 2.5. Selection of Prediction Models for External Validation

We performed a systematic review for external validation; a total of 524 articles were screened. The searching strategy is provided in Table S1. After manual review, we identified 21 candidate models for PTB prediction. We excluded four models which mainly focused on specific populations, eleven models with laboratory measures and/or ultrasound measures not routinely available in most developing countries, two models that could not be applied to early pregnancy, and one model that used continuous pregnancy length as the outcome. One of the remaining four prediction models eligible for external validation lacked information on the equation coefficient [21]. We contacted the author by email to obtain these but received no response. Finally, three models were validated in the present study [22,23,24]. The flow chart of model selection is shown in Figure S1. For the included models, we transformed the odds ratios of predictors to regression coefficients and obtained the equations of the models (Table S2). We then performed recalibration by fitting logistic regression models using the linear predictor as the only covariate. AUCs and calibration plots were constructed for the recalibration model using BIGCS data wherein Rubin’s rule was used to combine the AUCs and observed and predicted risks across imputed datasets.

## 3. Results

### 3.1. Characteristics of Participants

For the present study, we used data from 10,277 pregnant women recruited between February 2012 and February 2014 in BIGCS. Women with a multiple pregnancy (*n* = 212) or a pregnancy that occurred after assisted reproductive technology (*n* = 293) were not included. We also excluded women who withdrew before delivery (*n* = 435), terminated their pregnancies, or had stillbirths (*n* = 111), and women who had missing data on gestational age at delivery (*n* = 182). This resulted in an analytic sample of 9044 women.

Among the 9044 women with a singleton birth, 444 (4.9%) women had PTB. More than 83% of the PTBs were spontaneous. Tables S3–S6 shows the distribution of maternal characteristics of women who delivered preterm and at term. Older (Table S3) and multiparous women, women with a history of preterm delivery (Table S4), and women who had experienced vaginal bleeding during pregnancy (Table S5) had higher rates of overall, spontaneous and iatrogenic PTB. In contrast, women who reported use of folic acid before pregnancy had a lower rate of overall PTB (Table S6).

### 3.2. Discrimination of Models for Overall PTB

For the prediction of overall PTB, advanced maternal age, lower maternal height, history of preterm delivery, amount of vaginal bleeding during pregnancy, and lack of folic acid intake before pregnancy were important predictors for increased risk of PTB (Table 1). The highest risk was noted for history of preterm birth; women with a history of preterm birth had about four-fold risk of PTB compared to women who did not have a history of preterm birth. There was no substantial difference in the performance between Model 3 (Harrell’s c statistic (95% CI), 0.61 (0.59–0.64); D statistic (95% CI), 0.23 (0.20–0.25)), Model 2 (Harrell’s c statistic (95% CI), 0.61 (0.59–0.64); D statistic (95% CI), 0.23 (0.20–0.25)), and Model 1 (Harrell’s c statistic (95% CI), 0.59 (0.56–0.61); D statistic (95% CI), 0.18 (0.15–0.20)).

### 3.3. Discrimination of Models for Spontaneous and Iatrogenic PTB

The predictors of spontaneous PTB were similar to those for overall PTB, except for maternal height (Table 1). Model performance was almost the same as for overall PTB, with a Harrell’s c statistic (95% CI) of 0.60 (0.57–0.63) and a D statistic (95% CI) of 0.20 (0.18–0.23) in Model 3. For iatrogenic PTB, the final prediction model (Model 3) consisted of maternal age, amount of vaginal bleeding during pregnancy, and passive smoking during pregnancy (Table 1); the Harrell’s c statistic (95% CI) was 0.64 (0.61–0.66) and the D statistic (95% CI) 0.28 (0.26–0.31).

### 3.4. Discrimination of Models for Late and Early PTB

The important predictors for late PTB included maternal age, history of preterm delivery, and amount of vaginal bleeding during pregnancy. Furthermore, the final model for early PTB among nulliparous women included only maternal age, and for multiparous women the model included only the history of preterm delivery (Table 2). Multiparous women with a history of preterm birth had a six times higher risk for early PTB compared to multiparous women without a history of preterm birth. The final model for early PTB among multiparous women had a relatively higher Harrell’s c statistic (95% CI) (0.66 (0.63–0.68)) and D statistic (95% CI) (0.32 (0.30–0.35)) compared to that for early PTB (Harrell’s c statistic (95% CI): 0.60 (0.57–0.62); D statistic (95% CI): 0.20 (0.18–0.23)) among nulliparous women and for late PTB (Harrell’s c statistic (95% CI): 0.60 (0.58–0.63) and a D statistic (95% CI) of 0.21 (0.18–0.24)). 

### 3.5. Calibration Plots

**Figure 1:**
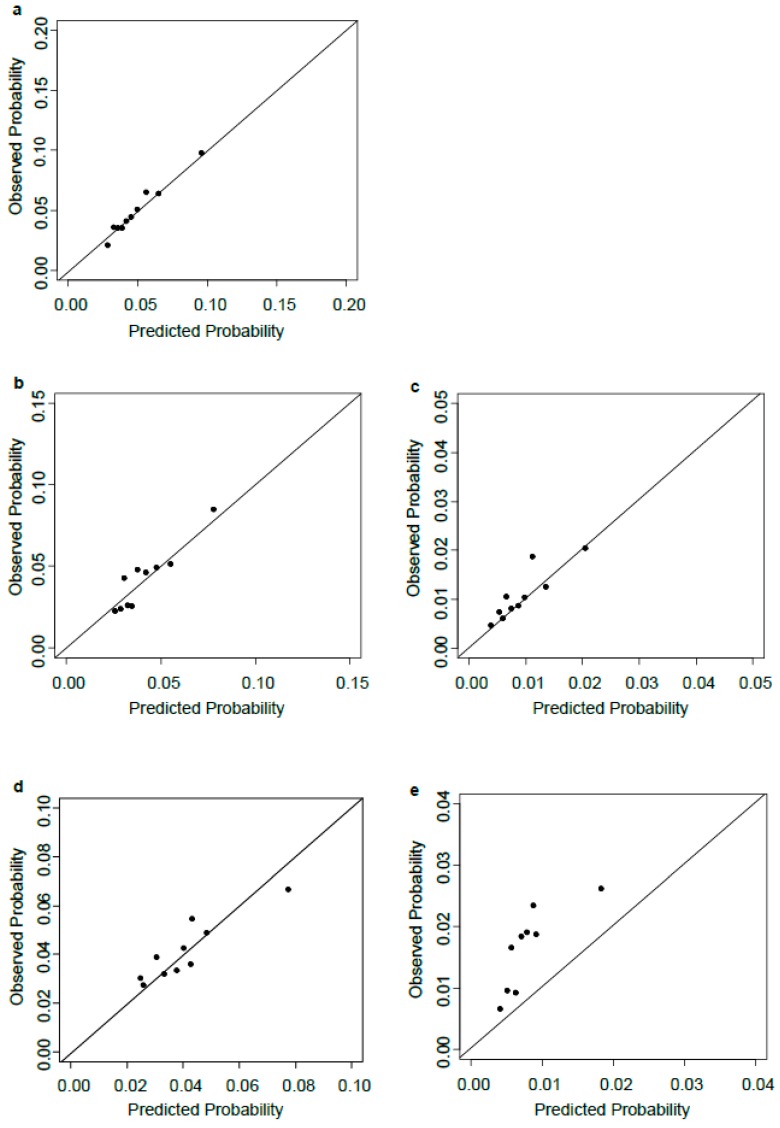
Calibration plots for the predicted probability and observed proportions of (**a**) overall preterm birth, (**b**) spontaneous preterm birth and (**c**) iatrogenic preterm birth, (**d**) late preterm birth, and (**e**) early preterm birth. The black dots indicate deciles of women with similar predicted risk of preterm birth.

Figure 1 illustrates the calibration plots for each model. Model fit was good for all models (*p* values for the Nam and D’Agostino test > 0.05) except the model for early PTB.

### 3.6. Models Stratified by Parity

Among nulliparous women, advanced maternal age, lower maternal height, amount of vaginal bleeding, and folic acid intake before pregnancy were predictors for overall PTB in the final model (Table S7). Among multiparous women, history of preterm birth was a strong predictor for overall PTB followed by family history of hypertension (Table S7). For spontaneous PTB, the predictors were the same as for overall PTB except for maternal height among nulliparous women. The model for overall PTB among nulliparous and multiparous women had Harrell’s c statistic (95% CI) of 0.60 (0.57–0.63) and 0.64 (0.61–0.66) and D statistics (95% CI) of 0.20 (0.18–0.23) and 0.28 (0.26–0.31), respectively. For the prediction model of spontaneous PTB, the model for multiparous women had a slightly higher Harrell’s c statistic (95% CI) (0.64 (0.62–0.67)) and D statistic (95% CI) (0.30 (0.27–0.32)) compared to the model for nulliparous women Harrell’s c statistic (95% CI) (0.59 (0.57–0.62)) and D statistic (95% CI) (0.19 (0.16–0.21)).

### 3.7. External Validation for Published Models

Finally, we validated three published prediction models [22,23,24]. The model for the prediction of spontaneous delivery at <34 weeks by Parra-Cordero M et al. [24] among multiparous women included only a history of preterm delivery, while the model for nulliparous women included only smoking during pregnancy. The model generated by Sananes et al. [22] included maternal age, BMI, smoking and obstetric history as predictors and spontaneous delivery <37 weeks as outcome with an AUC (95% CI) of 0.62 (0.60, 0.64) in original population. The model established by Beta et al. [23] used maternal age, height, racial origin, smoking, assisted conception, and obstetric history to predict spontaneous delivery at <34 weeks and had an AUC (95% CI) of 0.67 (0.64, 0.70) in original population. The AUCs of these three models in our population were 0.56 (0.51, 0.62), 0.50 (0.47, 0.53), and 0.54 (0.46, 0.62), respectively. The calibration plots showed that the predicted risks based on these three models did not closely match the observed frequencies (Figure S2).

## 4. Discussion

In the present study, we developed predictive models for overall PTB, spontaneous and iatrogenic PTB, and late and early PTB using maternal sociodemographic, behavioral, psychosocial, and medical characteristics during early pregnancy in a Chinese population. Overall, the performance of models was moderate. We also validated three published models that used easily available predictors and found that these models had poor performance in our population.

There are several strengths to our study. First, we used comprehensive data on maternal characteristics, including sociodemographic characteristics, obstetric history, disease history, behavioral factors, and psychological status. Second, the prospective design of our study enabled us to reduce recall bias to some extent, especially for the variables of behavioral factors and psychological status. Third, the sample size in the present study was large, which was roughly equal to the sum of the two previous Chinese studies combined [11,12]. Fourth, we used Cox proportional hazard model to construct the prediction models, while previous studies all used logistic models. Cox models are more suitable than logistic models for PTB prediction because preterm birth is a time-dependent event. Some limitations in the present study should be noted. We did not use data obtained from ultrasound examinations (e.g, cervical length) or laboratory testing (e.g., pregnancy-associated plasma protein A). Our intention was to use maternal characteristics that are readily available and relevant to clinical practice in limited-resource countries, including China. In these countries, ultrasound examinations and laboratory testing may not be affordable. Additionally, the validity of these tests in early pregnancy to predict the risk of PTB remains unclear [5,22]. Another limitation is, the absence of model validation in a separate population. Furthermore, we did not exclude women with a high risk for PTB and who might have received clinical interventions (e.g., progesterone administration). This might have reduced their risk for PTB thereby underestimating the predictive potential of our models. Finally, our data were collected from a single medical center (with two branches) and thus may not be representative of Guangzhou city or China.

Compared to prediction of PTB in late pregnancy, prediction using data available in the first or early second trimester has significant advantage in that it enables administration of preventive strategies or early treatment to prevent the occurrence of PTB. Several studies have explored predictive models for spontaneous PTB using data during early pregnancy [5]. Schaaf et al. used 13 predictors in the first or early second trimester to develop a predictive model in the Netherlands that had an AUC of 0.63 (95% CI 0.63–0.63) [25]. Their predictors included sociodemographic characteristics, obstetric and general disease history, and current pregnancy conditions [25]. Sananes et al. based their prediction model for spontaneous PTB on maternal factors and obstetric history using a French population [22]. Their final model had an AUC of 0.62; biomarkers in the first trimester of pregnancy could not improve the performance of this model [22]. In addition, a study conducted in the UK found that data on maternal characteristics and obstetric history at 11–13 weeks of gestation were predictive of spontaneous early preterm deliveries; this model had an AUC of 0.67 [23]. In the present study, we acquired easily accessible data on maternal characteristics obtained at recruitment using questionnaires. The potential predictors included sociodemographic characteristics, obstetric history, disease history, behavioral factors, and psychological status. Our models for PTB had Harrell’s c statistic ranging from 0.60 to 0.66, which was similar to the aforementioned studies [22,23,25]. A recent study also validated several models in a Dutch population and found poor to moderate model performance [26]. The moderate performance of these models suggests that prediction of PTB using accessible data on maternal characteristics and obstetric history at early pregnancy needs to be further improved.

Previous translational studies have found evidence linking previous pregnancy history to current pregnancy outcomes [27]. We found that the predictive ability of models was different for nulliparous and multiparous women. Specifically, the prediction model of overall PTB for multiparous women had a Harrell’s c statistic of 0.64 which was slightly better than the model for nulliparous women (Harrell’s c statistic, 0.60). This finding is consistent with results from previous studies [22,23,26]. It has been proposed that obstetric history of multiparous women could provide useful information about individual susceptibility to predict the occurrence of PTB in subsequent pregnancies [28,29]. In addition, most women who deliver prematurely have no obvious risk factors [6,30]. This might explain the difficulty to predict PTB among nulliparous women because limited information regarding risk factors (including obstetric history) can be used for prediction. 

There only two published prediction models of PTB that were developed using data from Chinese women. Leung et al. assessed the performance of a single cervical length measurement at mid-trimester to predict spontaneous preterm delivery and found the predictive ability was low in low-risk populations (AUC, 0.56) [12]. Xu et al. used seven factors to predict the occurrence of PTB, including abnormal uterine or uterine deformity, parity, number of pregnancies, gestational hypertension, placenta previa, premature rupture of membranes, and regular prenatal examination. Although this predictive model had a satisfactory AUC (about 0.8) [11], some variables in the prediction model were derived in late pregnancy, which limited the utility of the model for early pregnancy prediction. To the best of our knowledge, we are unaware of any published report regarding the prediction model of PTB using data obtained in early pregnancy in a Chinese population. 

## 5. Conclusions

We built predictive models for PTB using accessible data for maternal characteristics during early pregnancy in a Chinese population, albeit the performance of our models was moderate. Previously published models performed poorly in our population, which challenges their clinical applications. Further improvement in the predictive ability of PTB models using early pregnancy characteristics may require the inclusion of laboratory measures or biomarkers.

## Figures and Tables

**Table 1:** Variables in the prediction models for all preterm and spontaneous preterm births.

Predictors	HR (95% CI)		
	Model 1 ^1^	Model 2 ^2^	Model 3 ^3^
Models for all PTB			
Age (per year increase)	1.06 (1.03–1.08)	1.05 (1.02–1.08)	1.05 (1.02–1.08)
Height (per cm increase)	0.98 (0.96–1.00)	0.98 (0.96–1.00)	0.98 (0.96–1.00)
History of preterm delivery			
No	1.00 (reference)	1.00 (reference)	1.00 (reference)
Yes	3.97 (2.31–6.83)	4.01 (2.36–6.81)	3.95 (2.33–6.68)
Amount of vaginal-bleeding			
Never		1.00 (reference)	1.00 (reference)
Mild		1.66 (1.35–2.05)	1.66 (1.34–2.04)
Moderate or severe		1.46 (1.00–2.13)	1.47 (1.00–2.14)
Folic acid intake before pregnancy			
Never			1.00 (reference)
Ever			0.78 (0.64–0.95)
Models for spontaneous PTB			
Age (per year increase)	1.05 (1.02–1.08)	1.05 (1.02–1.08)	1.05 (1.02–1.08)
History of preterm delivery			
No	1.00 (reference)	1.00 (reference)	1.00 (reference)
Yes	4.09 (2.42–6.91)	4.06 (2.42–6.81)	4.00 (2.39–6.72)
Amount of vaginal-bleeding			
Never		1.00 (reference)	1.00 (reference)
Mild		1.64 (1.31–2.07)	1.64 (1.30–2.06)
Moderate or severe		1.71 (1.15–2.52)	1.71 (1.16–2.53)
Folic acid intake before pregnancy			
Never			1.00 (reference)
Ever			0.79 (0.63–0.98)
Models for iatrogenic PTB			
Age (per year increase)	1.09 (1.02–1.16)	1.09 (1.02–1.16)	1.09 (1.02–1.17)
Amount of vaginal-bleeding			
Never		1.00 (reference)	1.00 (reference)
Mild		1.69 (1.05–2.72)	1.69 (1.05–2.70)
Moderate or severe		0.41 (0.06–2.78)	0.41 (0.06–2.74)
Passive smoking during pregnancy			
Never			1.00 (reference)
Ever			1.75 (1.09–2.83)

PTB: preterm birth; HR, hazard ratio; CI, confidence interval. ^1^ Based on variables of maternal age, educational level, monthly income, height, pre-pregnancy body mass index, gravidity, parity, history of preterm delivery, and family history of diabetes or hypertension. ^2^ Based on variables in Model 1 and plus vaginal bleeding during pregnancy, anxiety and depression during pregnancy. ^3^ Based on variables in Model 2 and plus smoking during peri-conception period, passive smoking before pregnancy, passive smoking during pregnancy, folic acid intake before pregnancy and folic intake during pregnancy.

**Table 2:** Variables in the prediction models for late and early preterm births.

Predictors	HR (95% CI)		
	Model 1 ^1^	Model 2 ^2^	Model 3 ^3^
Models for PTB at 34–36 weeks			
Age (per year increase)	1.05 (1.02–1.08)	1.05 (1.02–1.08)	1.05 (1.02–1.08)
History of preterm delivery			
No	1.00 (reference)	1.00 (reference)	1.00 (reference)
Yes	2.81 (1.36–5.82)	2.82 (1.34–5.94)	2.82 (1.34–5.94)
Amount of vaginal-bleeding			
Never		1.00 (reference)	1.00 (reference)
Mild		1.65 (1.32–2.06)	1.65 (1.32–2.06)
Moderate or severe		1.44 (0.95–2.18)	1.44 (0.95–2.18)
Model for PTB at <34 weeks			
Nulliparous women			
Age (per year increase)	1.12 (1.04–1.20)	1.12 (1.04–1.20)	1.08 (1.01–1.16)
Multiparous women			
History of preterm delivery			
No	1.00 (reference)	1.00 (reference)	1.00 (reference)
Yes	6.25 (2.06–19.0)	6.25 (2.06–19.0)	6.25 (2.06–19.0)

PTB: preterm birth; HR, hazard ratio; CI, confidence interval. ^1^ Based on variables of maternal age, educational level, monthly income, height, pre-pregnancy body mass index, gravidity, parity, history of preterm delivery, and family history of diabetes or hypertension. ^2^ Based on variables retained in Model 1 plus vaginal bleeding during pregnancy, anxiety and depression during pregnancy. ^3^ Based on variables retained in Model 2 plus smoking during peri-conception period, passive smoking before pregnancy, passive smoking during pregnancy, folic acid intake before pregnancy and folic acid intake during pregnancy.

## Supplementary Materials

The following are available online at http://www.mdpi.com/2077-0383/7/8/185/s1, **Figure S1**: Flowchart of study inclusion in systematic review, **Figure S2**. Calibration plots for the predicted probability and observed proportions of models by (A) Beta J et al., (B) Sananes N et al., and (C) Parra-Cordero M et al. The black dots indicate deciles of women with similar predicted risk of preterm birth, **Table S1**: Search Strategy for MEDLINE, **Table S2**. Full equations for published model for preterm birth prediction, **Table S3**. Comparisons of socio-demographic characteristics between women with preterm and term births (before imputation), **Table S4**. Comparisons of obstetric and disease history between women with preterm and term births (before imputation), **Table S5**. Comparisons of pregnancy conditions between women with preterm and term births (before imputation), **Table S6**. Comparisons of modifiable factors between women with preterm and term births (before imputation), **Table S7**. Prediction models stratified by parity status.

## References

[1] Lawn J.E., Kinney M. (2014). Preterm birth: Now the leading cause of child death worldwide. Sci. Transl. Med..

[2] Liu L., Oza S., Hogan D., Perin J., Rudan I., Lawn J.E., Cousens S., Mathers C., Black R.E. (2015). Global, regional, and national causes of child mortality in 2000–13, with projections to inform post-2015 priorities: An updated systematic analysis. Lancet.

[3] Saigal S., Doyle L.W. (2008). An overview of mortality and sequelae of preterm birth from infancy to adulthood. Lancet.

[4] Kleinrouweler C.E., Cheong-See F.M., Collins G.S., Kwee A., Thangaratinam S., Khan K.S., Mol B.W.J., Pajkrt E., Moons K.G.M., Schuit E. (2016). Prognostic models in obstetrics: Available, but far from applicable. Am. J. Obstet. Gynecol..

[5] Sananes N., Langer B., Gaudineau A., Kutnahorsky R., Aissi G., Fritz G., Viville B., Nisand I., Favre R. (2014). Prediction of spontaneous preterm delivery in singleton pregnancies: Where are we and where are we going? A review of literature. J. Obstet. Gynaecol..

[6] Catley C., Frize M., Walker C.R., Petriu D.C. (2006). Predicting high-risk preterm birth using artificial neural networks. IEEE Trans. Inf. Technol. Biomed..

[7] Raglan G.B., Lannon S.M., Jones K.M., Schulkin J. (2016). Racial and Ethnic Disparities in Preterm Birth Among American Indian and Alaska Native Women. Matern. Child Health J..

[8] Culhane J.F., Goldenberg R.L. (2011). Racial disparities in preterm birth. Semin. Perinatol..

[9] York T.P., Strauss J.F., Neale M.C., Eaves L.J. (2010). Racial differences in genetic and environmental risk to preterm birth. PLoS ONE.

[10] Zou L., Wang X., Ruan Y., Li G., Chen Y., Zhang W. (2014). Preterm birth and neonatal mortality in China in 2011. Int. J. Gynaecol. Obstet..

[11] Xu X., Tan H., Zhou S., He Y., Shen L., Liu Y., Hu L., Wang X., Li X. (2014). Study on the application of Back-Propagation Artificial Neural Network used the model in predicting preterm birth. Zhonghua Liu Xing Bing Xue Za Zhi.

[12] Leung T.N., Pang M.W., Leung T.Y., Poon C.F., Wong S.M., Lau T.K. (2005). Cervical length at 18–22 weeks of gestation for prediction of spontaneous preterm delivery in Hong Kong Chinese women. Ultrasound Obstet. Gynecol..

[13] He J.R., Yuan M.Y., Chen N.N., Lu J.H., Hu C.Y., Mai W.B., Zhang R., Pan Y., Qiu L., Wu Y. (2015). Maternal dietary patterns and gestational diabetes mellitus: A large prospective cohort study in China. Br. J. Nutr..

[14] Qiu X., Lu J.H., He J.R., Lam K.H., Shen S.Y., Guo Y., Kuang Y.S., Yuan M.Y., Qiu L., Chen N.N. (2017). The Born in Guangzhou Cohort Study (BIGCS). Eur. J. Epidemiol..

[15] Zung W.W. (1965). A Self-Rating Depression Scale. Arch. Gen. Psychiatry.

[16] Zung W.W. (1971). A rating instrument for anxiety disorders. Psychosomatics.

[17] Goldenberg R.L., Culhane J.F., Iams J.D., Romero R. (2008). Epidemiology and causes of preterm birth. Lancet.

[18] Newson R.B. (2010). Comparing the predictive powers of survival models using Harrell’s C or Somers’ D. Stata J..

[19] Schuit E., Amer-Wahlin I., Groenwold R.H., Mol B.W., Moons K.G., Kwee A. (2012). Prediction of neonatal metabolic acidosis in women with a singleton term pregnancy in cephalic presentation: An external validation study. Am. J. Perinatol..

[20] Demler O.V., Paynter N.P., Cook N.R. (2015). Tests of calibration and goodness-of-fit in the survival setting. Stat. Med..

[21] Luo W., Huning E.Y., Tran T., Phung D., Venkatesh S. (2016). Screening for post 32-week preterm birth risk: How helpful is routine perinatal data collection?. Heliyon.

[22] Sananes N., Meyer N., Gaudineau A., Aissi G., Boudier E., Fritz G., Viville B., Nisand I., Langer B., Favre R. (2013). Prediction of spontaneous preterm delivery in the first trimester of pregnancy. Eur. J. Obstet. Gynecol. Reprod. Biol..

[23] Beta J., Akolekar R., Ventura W., Syngelaki A., Nicolaides K.H. (2011). Prediction of spontaneous preterm delivery from maternal factors, obstetric history and placental perfusion and function at 11–13 weeks. Prenat. Diagn..

[24] Parra-Cordero M., Sepulveda-Martinez A., Rencoret G., Valdes E., Pedraza D., Munoz H. (2014). Is there a role for cervical assessment and uterine artery Doppler in the first trimester of pregnancy as a screening test for spontaneous preterm delivery?. Ultrasound Obstet. Gynecol..

[25] Schaaf J.M., Ravelli A.C., Mol B.W., Abu-Hanna A. (2012). Development of a prognostic model for predicting spontaneous singleton preterm birth. Eur. J. Obstet. Gynecol. Reprod. Biol..

[26] Meertens L.J., van Montfort P., Scheepers H.C., van Kuijk S.M., Aardenburg R., Langenveld J., van Dooren I.M.A., Zwaan I.M., Spaanderman M.E.A., Smits L.J.M. (2018). Prediction models for the risk of spontaneous preterm birth based on maternal characteristics: A systematic review and independent external validation. Acta Obstet. Gynecol. Scand..

[27] Jayaram A., Kanninen T., Sisti G., Inglis S.R., Morgan N., Witkin S.S. (2017). Pregnancy History Influences the Level of Autophagy in Peripheral Blood Mononuclear Cells From Pregnant Women. Reprod. Sci..

[28] Morken N.H., Kallen K., Jacobsson B. (2014). Predicting risk of spontaneous preterm delivery in women with a singleton pregnancy. Paediatr. Perinat. Epidemiol..

[29] Kazemier B.M., Buijs P.E., Mignini L., Limpens J., de Groot C.J., Mol B.W. (2014). Impact of obstetric history on the risk of spontaneous preterm birth in singleton and multiple pregnancies: a systematic review. BJOG.

[30] Iams J.D., Goldenberg R.L., Mercer B.M., Moawad A.H., Meis P.J., Das A.F., Caritis S.N., Miodovnik M., Menard M.K., Thurnau G.R. (2001). The preterm prediction study: can low-risk women destined for spontaneous preterm birth be identified?. Am. J. Obstet. Gynecol..

