# Supplementary material for: Predictions of Preterm Birth from Early Pregnancy Characteristics: Born in Guangzhou Cohort Study
Source: J Clin Med. 2018 Jul 27;7(8):185. doi: 10.3390/jcm7080185 (PMC6111770; doi:10.3390/jcm7080185)
Supplement: Supplementary file 1 [file jcm-07-00185-s001.pdf]

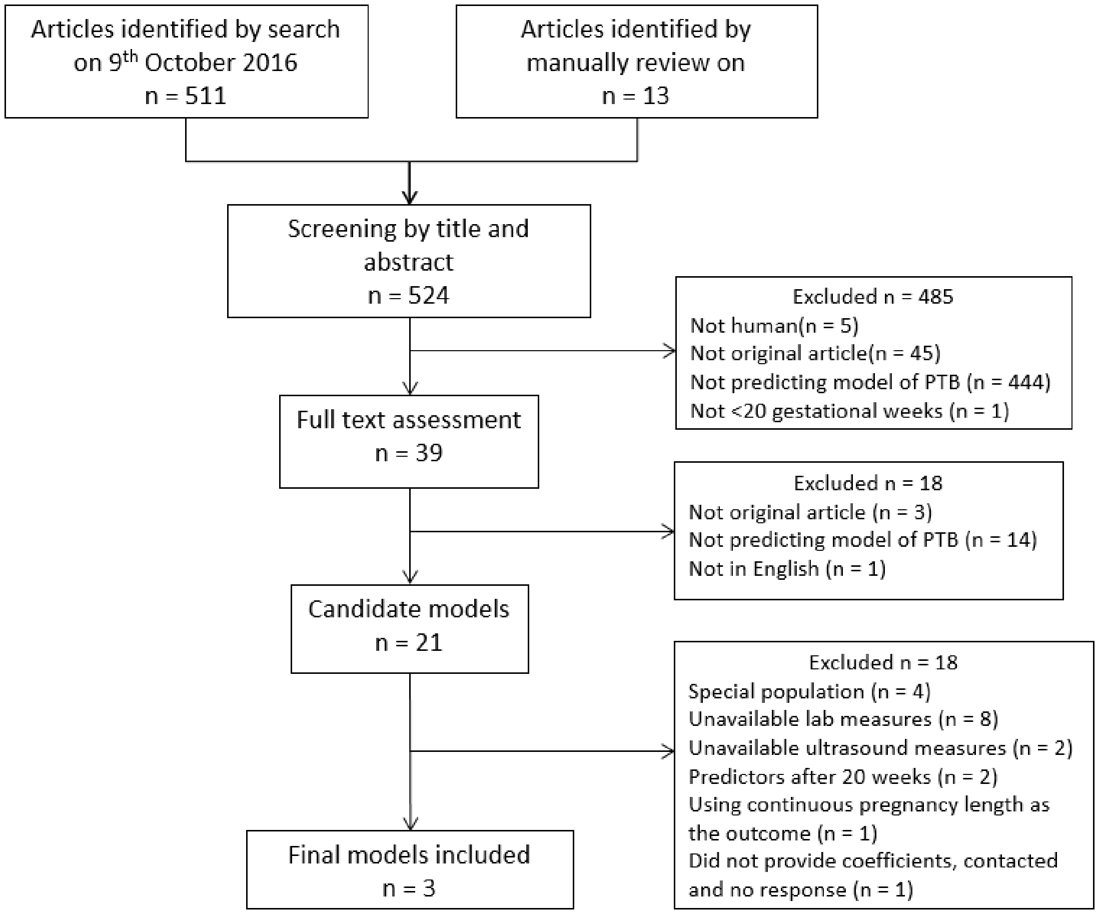

Figure S1. Flowchart of study inclusion in systematic review.

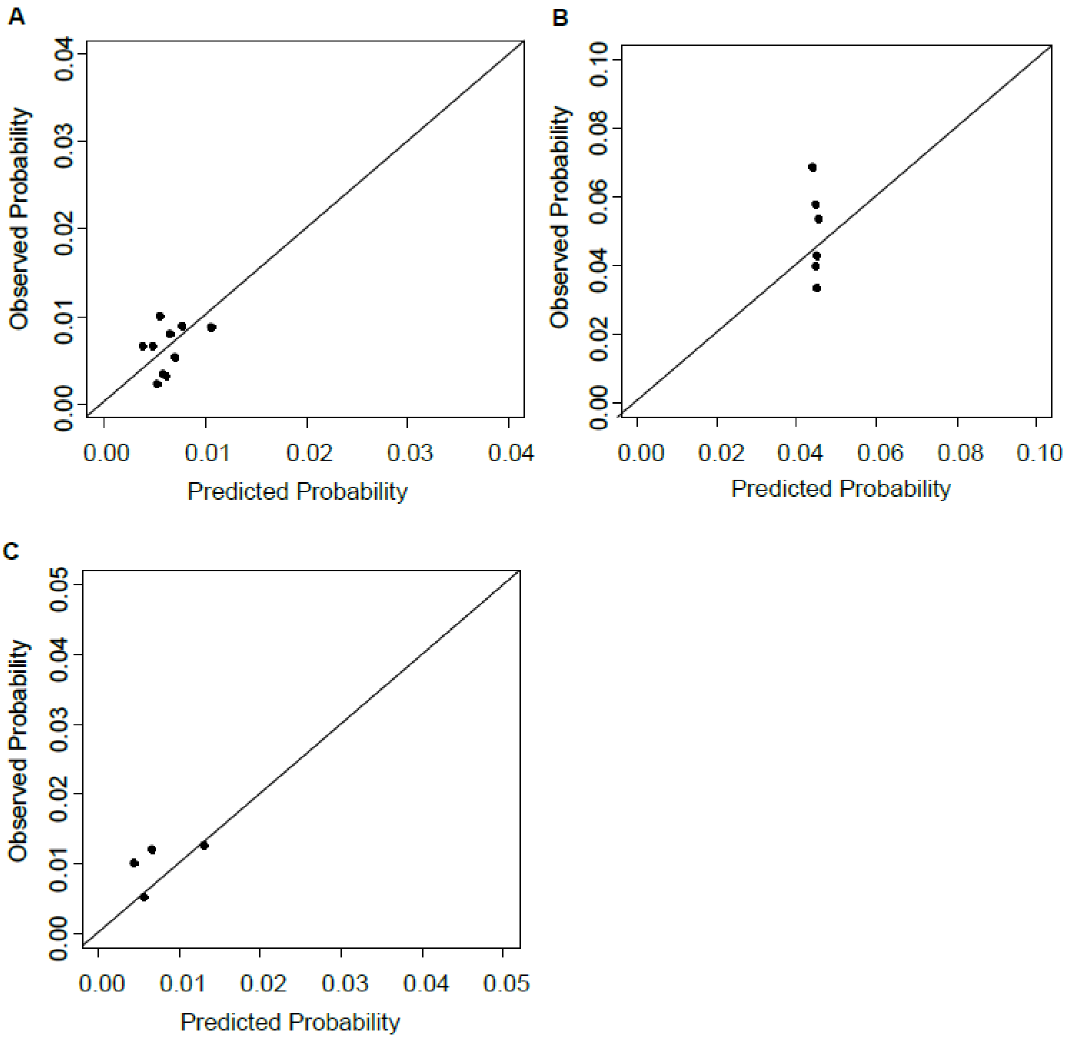

Figure S2. Calibration plots for the predicted probability and observed proportions of models by (A) Beta J *et al*., (B) Sananes N *et al.*, and (C) Parra-Cordero M et al. The black dots indicate deciles of women with similar predicted risk of preterm birth.

Table S1. Search Strategy for MEDLINE (adopted and modified from Am J Obstet Gynecol. 2016 Jan; 214(1):79-90 [1]).

Steps	Search term
#1	Search (Validat*[tiab] OR Predict*[ti] OR Rule*[tiab])
#2	Search (Predict*[tiab] AND (Outcome*[tiab] OR Risk*[tiab] OR Model*[tiab]))
#3	Search ((History[tiab] OR Variable*[tiab] OR Criteria[tiab] OR Scor*[tiab] OR Characteristic*[tiab] OR Finding*[tiab] OR Factor*[tiab]) AND (Predict*[tiab] OR Model*[tiab] OR Decision*[tiab] OR Identif*[tiab] OR Prognos*[tiab]))
#4	Search (Decision*[tiab] AND (Model*[tiab] OR Clinical*[tiab] OR Logistic Model*[tiab]))
#5	Search (Prognostic[tiab] AND (History[tiab] OR Variable*[tiab] OR Criteria[tiab] OR Scor*[tiab] OR Characteristic*[tiab] OR Finding*[tiab] OR Factor* [tiab] OR Model*[tiab]))
#6	Search (“risk score”[All fields] OR “prediction model”[All fields] OR “prediction rule”[All fields] OR “risk assessment”[All fields] OR “algorithm”[All fields])
#7	#1 OR #2 OR #3 OR #4 OR #5 OR #6
#8	Search (premature deliver*[tiab] OR premature labour[tiab] OR premature labor[tiab] OR premature birth[tiab] OR preterm deliver*[tiab] OR preterm labour[tiab] OR preterm labor[tiab] OR preterm birth[tiab])
#9	Search (first trimester[tiab] OR early pregnancy[tiab])
#10	#7 AND #8 AND #9

Table S2. Full equations for published model for preterm birth prediction.

Parra-Cordero M *et al.* (2014) [22]
The probability of preterm birth was calculated as: $e^X/(1+e^X)$, where<br>For Parous women, X= α + 1.52606 (if previous preterm delivery).<br>For nulliparous = α + 1.16315 (if smoking during pregnancy).
Sananes N *et al.* (2013) [20]
The probability of preterm birth was calculated as: $e^X/(1+e^X)$, where<br>X=α + 0.24920 × (if maternal age ≤22 or ≥35) + 0.32930 × (if BMI ≤ 19) + 0.28818 × (if smoke) + 0.77242 × (if previous miscarriage at 16–23 weeks) + 1.62492 × (if previous preterm delivery at 24–27 weeks) + 0.66217 × (if previous preterm delivery at 28–33 weeks) + 1.13269 × (if previous preterm delivery at 34–36 weeks) − 0.61990 × (if previous term delivery at ≥37 weeks).
Beta J *et al.* (2011) [21]
The probability of preterm birth was calculated as: $e^X/(1+e^X)$, where<br>X=α + 0.02469 × (maternal age, years) − 0.01918 × (height, cm) + 0.58945 (if African) + 0.55389 (if South Asian) + 0.16805 (if Eastern Asian) [#] − 0.40048 (if Mixed)+ 0.56702 × (if smoke) + 0.53532 (if assisted conception) [$] + 0.23902 × (if nulliparous and previous fetal loss at < 16 weeks) + 1.97602 × (if nulliparous and previous miscarriage at 16–23 weeks) + 1.73431 × (if nulliparous and previous miscarriage at 24–27 weeks) [&] + 1.50319 × (if previous preterm delivery at 28–30 weeks) [&] + 1.14199 × (if previous preterm delivery at 31–33 weeks) [&] + 0.90745 × (if previous preterm delivery at 34–36 weeks) [&] − 0.41400 × (if previous term delivery at >37 weeks) + 0.30895 (if parous iatrogenic preterm delivery) [&].

[#] All women are eastern Asian in the current study.

[$] All women had no assisted conception in the current study.

[&] These terms were generated using gestational age of previous delivery because we couldn’t differentiate if previous preterm delivery was spontaneous or not; therefore, we did not include the term, “if parous iatrogenic preterm delivery”, which was not significant in the original model (P = 0.60).

Table S3. Comparisons of socio-demographic characteristics between women with preterm and term births (before imputation).

Maternal characteristics	Term births, N (%)	Overall PTB		Spontaneous PTB		Iatrogenic PTB	
		N (%)	P	N (%)	P	N (%)	P
Total	8600	444		340		68	
Age (years), mean ± SD	28.7 ± 3.3	29.5 ± 3.4	<0.001	29.4 ± 3.5	<0.001	29.9 ± 3.2	0.01
Educational level							
Middle school or below	908	56 (5.8)	0.22	40 (4.2)	0.36	9 (1.0)	0.82
College	2205	126 (5.4)		99 (4.3)		18 (0.8)	
Undergraduate	4496	214(4.5)		163 (3.5)		35 (0.8)	
Postgraduate	991	48 (4.6)		38 (3.7)		6 (0.6)	
Monthly income (Yuan)							
≤1500	806	46 (5.4)	0.63	31 (3.7)	0.92	8 (1.0)	0.35
1501–4500	2620	124 (4.5)		99 (3.6)		14 (0.5)	
4501–9000	3403	171 (4.8)		130 (3.7)		30 (0.9)	
≥9001	1281	71 (5.3)		54 (4.0)		12 (0.9)	
Missing	490	32		26		4	
Height (cm), mean±SD	160.0 ± 4.8	159.5 ± 4.4	0.01	159.5 ± 4.5	0.07	159.3 ± 4.2	0.15
Missing	324	24		20		2	
Pre-pregnancy BMI (kg/m^2), mean±SD	20.3 ± 2.7	20.5 ± 2.8	0.08	20.5 ± 2.8	0.06	20.4 ± 3.1	0.46
Missing	439	32		28		2	

PTB: preterm birth; BMI: body mass index. P values based on t test for continuous variables and chi-square test for categorical variables. The total for spontaneous and iatrogenic births does not add to total preterm births due to missing values for these variables.

Table S4. Comparisons of obstetric and disease history between women with preterm and term births (before imputation).

Maternal characteristics	Term births, N	PTB		Spontaneous PTB		Iatrogenic PTB	
		N (%)	P	N (%)	P	N (%)	P
Obstetric history							
Gravidity							
1	5216	258 (4.7)	0.51	203 (3.8)	0.88	39 (0.7)	0.51
>1	3060	162 (5.0)		117 (3.7)		27 (0.9)	
Missing	324	24		20		2	
Parity							
Nulliparous	7608	376 (4.7)	0.02	290 (3.6)	0.12	58 (0.7)	0.37
Multiparous	962	65 (6.3)		47 (4.6)		10 (1.0)	
Missing	30	3		3		0	
History of previous preterm birth							
No	8414	419 (4.7)	<0.001	321 (3.6)	<0.001	65 (0.7)	0.01
Yes	50	12 (19.4)		10 (16.1)		2 (3.2)	
Missing	136	13		9		1	
Planned pregnancy							
Yes	6222	310 (4.8)	0.59	239 (3.7)	0.85	48 (0.8)	0.75
No	2134	113 (5.0)		84 (3.8)		18 (0.8)	
Missing	244	21		17		2	
History of disease							
Diabetes/hypertension before pregnancy							

No	8267	419 (4.8)	0.84	319 (3.7)	0.64	66 (0.8)	0.72
Yes	16	1 (5.9)		1 (5.9)		0 (0)	
Missing	317	24		20		2	
Thyroid disease before pregnancy							
Never	8129	413 (4.8)	0.28	314 (3.7)	0.51	65 (0.8)	0.35
Only hypothyroidism	43	4 (8.5)		3 (6.5)		1 (2.3)	
Only hyperthyroidism or both	110	3 (2.7)		3 (2.7)		0 (0)	
Missing	318	24		20		2	
Family history of diabetes							
No	7510	375 (4.8)	0.37	284 (3.6)	0.23	60 (0.8)	0.80
Yes	835	48 (5.4)		39 (4.5)		6 (0.7)	
Missing	255	21		17		2	
Family history of hypertension							
No	6161	303 (4.7)	0.30	231 (3.6)	0.32	48 (0.8)	0.85
Yes	2192	121 (5.2)		93 (4.1)		18 (0.8)	
Missing	247	20		16		2	
Family history of heart disease							
No	7948	400 (4.8)	0.35	305 (3.7)	0.32	62 (0.8)	0.59
Yes	389	24 (5.8)		19 (4.7)		4 (1.0)	
Missing	263	20		16		2	

PTB: preterm birth. P values were derived from chi-square tests

The total for spontaneous and iatrogenic births does not add to total preterm births due to missing values for these variables.

Table S5. Comparisons of pregnancy conditions between women with preterm and term births (before imputation).

Maternal characteristics	Term births N (%)	PTB		Spontaneous PTB		Iatrogenic PTB	
		N (%)	P	N (%)	P	N (%)	P
Timing of vaginal bleeding							
Never	5937	251 (4.1)	<0.001	190 (3.1)	<0.001	40 (0.7)	0.03
<13 weeks	2087	133 (6.0)		107 (4.9)		18 (0.9)	
≥13 weeks	227	26 (10.3)		19 (7.7)		5 (2.2)	
Missing	349	24		24		5	
Amount of vagina bleeding							
Never	5937	251 (4.1)	<0.001	190 (3.1)	<0.001	40 (0.7)	0.02
Mild	1948	139 (6.7)		108 (5.3)		24 (1.2)	
Moderate or severe	450	30 (6.3)		24 (5.1)		1 (0.2)	
Missing	265	24		18		3	
Anxiety during pregnancy	41.7 ± 7.4	41.9 ± 7.6	0.61	41.7 ± 7.6	0.66	43.1 ± 7.8	0.22
Missing	402	28		24		3	
Depression during pregnancy	44.5 ± 8.8	44.5 ± 9.1	0.93	44.5 ± 9.2	0.72	45.2 ± 9.5	0.98
Missing	402	28		24		2	

PTB: preterm birth. P values were derived from chi-square tests.

The total for spontaneous and iatrogenic births does not add to total preterm births due to missing values for these variables.

Table S6. Comparisons of modifiable factors between women with preterm and term births (before imputation).

Maternal characteristics	Term births, N	PTB		Spontaneous PTB		Iatrogenic PTB	
		N (%)	P	N (%)	P	N (%)	P
Working posture							
Not work	1148	63 (5.2)	0.26	44 (3.7)	0.83	11 (1.0)	0.21
Sitting	5075	245 (4.6)		196 (3.7)		33 (0.7)	
Standing	1890	108 (5.4)		77 (3.9)		21 (1.1)	
Walking and others	247	8 (3.1)		7 (2.8)		1 (0.4)	
Missing	240	20		16		2	
Smoking during peri-conception period							
Never	8156	412 (4.8)	0.71	314 (3.7)	0.86	64 (0.8)	0.29
Ever	120	7 (5.5)		5 (4.0)		2 (1.6)	
Missing	324	25		21		2	
Passive smoking before pregnancy							
Never	5195	254 (4.7)	0.37	203 (3.8)	0.76	35 (0.7)	0.10
Ever	3077	165 (5.1)		116 (3.6)		31 (1.0)	
Missing	328	25		21		2	
Passive smoking during pregnancy							
Never	5748	288 (4.8)	0.80	228 (3.8)	0.40	39 (0.7)	0.07
Ever	2523	130 (4.9)		90 (3.4)		27 (1.1)	
Missing	329	26		22		2	

Folic acid intake before pregnancy							
Never	4627	259 (5.3)	0.02	193 (4.0)	0.11	42 (0.9)	0.21
Ever	3645	160 (4.2)		126 (3.3)		24 (0.7)	
Missing	328	25		21		2	
Folic acid intake during pregnancy							
Never	723	35 (4.6)	0.79	28 (3.7)	0.98	2 (0.3)	0.10
Ever	7555	384 (4.8)		291 (3.7)		64 (0.8)	
Missing	322	25		21		2	

PTB: preterm birth. P values were derived from chi-square tests.

The total for spontaneous and iatrogenic births does not add to total preterm births due to missing values for these variables.

Table S7. Prediction models stratified by parity status.

Predictors	Nulliparous women		Multiparous women	
	**HR (95% CI)**	**P**	**HR (95% CI)**	**P**
**Models for all PTB**				
Age (per year increase)	1.07 (1.03–1.10)	<0.001	-	
Height (per cm increase)	0.98 (0.96–1.00)	0.03	-	
Amount of vaginal-bleeding				
Never	1.00 (reference)		-	
Mild	1.77 (1.41–2.21)	<0.001	-	
Moderate or severe	1.63 (1.09–2.44)	0.02	-	
Folic acid intake before pregnancy				
Never	1.00 (reference)		-	
Ever	0.79 (0.64–0.97)	0.03	-	
History of previous preterm birth				
No	-		1.00 (reference)	<0.001
Yes	-		4.84 (2.57–9.15)	
Family history of hypertension				
No	-		1.00 (reference)	0.02
Yes	-		1.81 (1.08–3.03)	
**Models for spontaneous PTB**				
Age (per year increase)	1.06 (1.02–1.09)	0.001		
Amount of vaginal-bleeding				
Never	1.00 (reference)			
Mild	1.79 (1.40–2.29)	<0.001		
Moderate or severe	1.90 (1.26–2.89)	0.002		
Folic acid intake before pregnancy				
Never	1.00 (reference)	0.04		
Ever	0.78 (0.62–0.99)			
History of previous preterm birth				
No	-		1.00 (reference)	<0.001
Yes	-		4.93 (2.58–9.42)	
Family history of hypertension				
No	-		1.00 (reference)	0.02
Yes	-		1.98 (1.12–3.51)	
**Models for iatrogenic PTB [a]**				
Age (per year increase)	1.11 (1.04–1.19)	0.004	-	

PTB: preterm birth. HR: hazard ratios. CI: confidence interval.

[a] There was no significant predictor for multiparous women.

References

1. Kleinrouweler, C.E.; Cheong-See, F.M.; Collins, G.S.; Kwee, A.; Thangaratinam, S.; Khan, K.S.; Mol, B.W.J.; Pajkrt, E.; Moons, K.G.M.; Schuit, E. Prognostic models in obstetrics: Available, but far from applicable. *Am. J. Obstet. Gynecol.* **2016**, *214*, 79–90 e36, doi:10.1016/j.ajog.2015.06.013.
